# Physiologic effects of alveolar recruitment and inspiratory pauses during moderately-high-frequency ventilation delivered by a conventional ventilator in a severe lung injury model

**DOI:** 10.1371/journal.pone.0185769

**Published:** 2017-09-29

**Authors:** Ricardo Luiz Cordioli, Eduardo Leite Vieira Costa, Luciano Cesar Pontes Azevedo, Susimeire Gomes, Marcelo Britto Passos Amato, Marcelo Park

**Affiliations:** 1 Research and Education Institute, Hospital Sírio-Libanês, São Paulo, Brazil; 2 Cardio-Pulmonary Department, Pulmonary Division, Heart Institute (Incor), University of São Paulo, São Paulo, Brazil; 3 Intensive Care Unit, Hospital Israelita Albert Einstein, São Paulo, Brazil; 4 Intensive Care Unit, Hospital Alemão Oswaldo Cruz, São Paulo, Brazil; 5 Emergency Medicine Discipline, Universidade de São Paulo, São Paulo, Brazil; University of Bari, ITALY

## Abstract

**Background and aims:**

To investigate whether performing alveolar recruitment or adding inspiratory pauses could promote physiologic benefits (VT) during moderately-high-frequency positive pressure ventilation (MHFPPV) delivered by a conventional ventilator in a porcine model of severe acute respiratory distress syndrome (ARDS).

**Methods:**

Prospective experimental laboratory study with eight pigs. Induction of acute lung injury with sequential pulmonary lavages and injurious ventilation was initially performed. Then, animals were ventilated on a conventional mechanical ventilator with a respiratory rate (RR) = 60 breaths/minute and PEEP titrated according to ARDS Network table. The first two steps consisted of a randomized order of inspiratory pauses of 10 and 30% of inspiratory time. In final step, we removed the inspiratory pause and titrated PEEP, after lung recruitment, with the aid of electrical impedance tomography. At each step, PaCO_2_ was allowed to stabilize between 57–63 mmHg for 30 minutes.

**Results:**

The step with RR of 60 after lung recruitment had the highest PEEP when compared with all other steps (17 [16,19] vs 14 [10, 17]cmH_2_O), but had lower driving pressures (13 [13,11] vs 16 [14, 17]cmH_2_O), higher P/F ratios (212 [191,243] vs 141 [105, 184] mmHg), lower shunt (23 [20, 23] vs 32 [27, 49]%), lower dead space ventilation (10 [0, 15] vs 30 [20, 37]%), and a more homogeneous alveolar ventilation distribution. There were no detrimental effects in terms of lung mechanics, hemodynamics, or gas exchange. Neither the addition of inspiratory pauses or the alveolar recruitment maneuver followed by decremental PEEP titration resulted in further reductions in VT.

**Conclusions:**

During MHFPPV set with RR of 60 bpm delivered by a conventional ventilator in severe ARDS swine model, neither the inspiratory pauses or PEEP titration after recruitment maneuver allowed reduction of VT significantly, however the last strategy decreased driving pressures and improved both shunt and dead space.

## Introduction

Acute respiratory distress syndrome (ARDS) is a common cause of mortality and morbidity in critically ill patients [1]. Although, crucial in the support of ARDS patients, mechanical ventilation comprises the application of mechanical forces to the lung parenchyma that can further induce injury to the lungs [2], adding morbidity and mortality [3].

Lowering tidal volume (VT) during ventilation, even below 6mL/kg of predicted body weight, decreases the cyclic stretch imposed on the lung [4, 5]. Conversely, low VT ventilation has the potential to lead to clinically significant hypercapnia-related acidosis, a potentially life-threatening condition. In this scenario, especially in more severe lung injury, high-frequency oscillatory ventilation (HFOV) has been tested, in order to provide adequate gas exchange even at very low VT. Early studies on this technique had promising results [6–8] but results of more recent trials were disappointing [9, 10]. Some explanations might be that the technique is requires the use of high airway pressures, which may have deleterious effects, especially on the right ventricle [11, 12]. A recent study published by Amato et al which analyzed the data from 3562 patients with ARDS enrolled in nine previously reported randomized trials. The driving pressure was the ventilation variable that best stratified the risk of mortality even more than the tidal volume did. Decreases in driving pressure were strongly associated with increased survival [13].

An alternative approach would be to apply moderately-high-frequency positive pressure ventilation (MHFPPV) using conventional mechanical ventilators [11]. This strategy employs intermediate respiratory rates between those used conventionally (≤ 35 min^-1^) and those used during HFOV (180–800 min^-1^) and has been shown to be effective and safe in a swine model of ARDS^11^ and in patients as well [14]. The respiratory rates during MHFPPV are variably limited by the occurrence of intrinsic positive end-expiratory pressure (PEEPi), depending on the mechanical properties of the respiratory system. Additionally, as VT approaches the dead space, especially at rates higher than 90 min^-1^, the reductions in VT tend not to be significant. Consequently, using this technique, further lowering of VT would require minimization of dead space.

We hypothesized that inspiratory pauses and/or alveolar recruitment maneuvers would promote reductions in dead space during MHFPPV. If accomplished, the lower dead space would lead to additional reductions in the VT while maintaining acceptable levels of carbon dioxide (CO_2_). The addition of an inspiratory pause reduces anatomical dead space by allowing CO_2_ to diffuse into the airways [15, 16], and also promotes better ventilation/perfusion (V/Q) matching at the alveolar level [17]. Recruitment maneuvers, however, can be a double-edged sword in terms of dead space. They have the potential to reduce shunt dead space [18], but could also increase the alveolar dead space due to compression of capillaries caused by the high positive airway pressures. Other settings can influence the tidal volume during high-frequency ventilation [19].

The aim of this study was to investigate whether alveolar recruitment and / or an addition of an inspiratory pause associated with MHPPV may further reduce the VT, maintaining PaCO_2_ at stable levels and consequently promoting other physiological benefits such as reduction of respiratory system pressures.

## Methods

This study was approved by the Institutional Animal Research Ethics Committees of Hospital Sírio Libanês and of Faculdade de Medicina da Universidade de São Paulo, both in São Paulo (CEUA 270/11)–Brazil, and was performed according to the National Institutes of Health (USA) guidelines for the use of experimental animals.

### Instrumentation

Previously healthy male Agroceres^®^ pigs were fasted overnight before the experiment with free access to water. Anesthesia and instrumentation were performed as described before [11]. Briefly, pre-anesthesia was done with an intramuscular injection of midazolam (0.3 mg/kg; Dormonid^®^, Roche, Brazil) and acepromazine (0.5 mg/kg; Acepran^®^, Andrômaco, Brazil). Anesthesia was induced with thionembutal (12 mg/kg; Tiopental^®^, Abbott, Brazil) and muscular relaxation with pancuronium bromide (0.1 mg/kg; Pavulon^®^, AKZO Nobel, Brazil). The animals were then intubated and connected to the Servo-300 mechanical ventilator (Maquet, Rastatt, Germany) with the following parameters in volume controlled mode: VT of 8–10 mL/kg, positive end-expiratory pressure (PEEP) of 5 cmH_2_O, inspiratory fraction of oxygen (FiO_2_) adjusted to keep arterial saturation between 94 and 96%, and respiratory rate (RR) necessary to keep the partial pressure of carbon dioxide (PaCO_2_) between 35 and 45 mmHg. Anesthesia was maintained during the study period with midazolam (0.3 mg/kg/h), fentanyl citrate (5 mcg/kg/h; Fentanyl^®^, Janssen-Cilag, Brazil), and muscular relaxation with pancuronium bromide (0.2 mg/kg/h). The adequate depth of anesthesia during the entire experiment was evaluated with maintenance of physiological variables (heart rate and arterial pressure). The absence of reflexes (corneal and hind limb flexion response), as well as unresponsiveness to stimuli during manipulation was also frequently tested, mainly before pancuronium infusion beginning. A continuous drip of 1000mL/h of Lactated Ringer solution was infused until the end of the induction of pulmonary injury, and then a continuous infusion of 5 ml/kg/h of Lactated Ringer was maintained until the end of the study.

Monitoring with continuous electrocardiography, oximetry, and blood pressures were done with a multiparameter monitor (Dixtal-Phillips DX 2020, São Paulo, Brazil). The left femoral artery was cannulated for blood pressure monitoring and blood samples acquisition. The right internal jugular vein was cannulated with a 9-French introducer sheath (Arrow, Reading, PA, USA) through which a pulmonary artery catheter (Edwards Lifesciences, Irvine, CA, USA) was introduced for monitoring of the mean pulmonary artery pressure (PAPm), cardiac output, central venous pressure (CVP) and mixed venous blood gases (SvO_2_). A central venous catheter was introduced in the left internal jugular vein. A surgical cystostomy was done to quantify the urine output. The animal was connected to the NICO_2_ device (Novametrix Medical Systems, Wallingford, Connecticut, USA) for end-tidal pressure of carbon dioxide (EtCO_2_), tidal volume, airway pressures, and airway flow monitoring.

The regional ventilation was monitored with the electrical impedance tomography (EIT) (Dixtal-Phillips, São Paulo, Brazil). The lungs were split in ventral and dorsal regions of same height. The amount of ventilation to the regions studied was reported according to the ventilatory mode used. Arterial blood gases analyses were done with the ABL 700 device (Radiometer, Copenhagen, Denmark). After the surgical period, the animals were allowed to rest for 60 minutes prior to the baseline data acquisition.

### Measurements

In all the steps of the study, the following data were collected:

1. Hemodynamic: heart rate, cardiac output, CVP, mean systemic arterial blood pressure (ABPm), PAPm, pulmonary artery occlusion pressure (PAOP), SvO_2_ and norepinephrine use and dosage.
2. Respiratory: partial pressure of arterial oxygen (PaO_2_), PaCO_2_, EtCO_2_, VT, airway peak pressure (Ppeak), airway plateau pressure (Pplateau) through the expiratory valve occlusion and two seconds of inspiratory pause, intrinsic positive end-expiratory pressure (PEEPi) through the expiratory valve occlusion after four seconds of expiratory pause, extrinsic positive end-expiratory pressure (PEEPe), mean airway pressure (Pmean), inspiratory flow, inspiratory time (T_insp_) and ventilatory distribution EIT data.
3. Metabolic: pH, lactate, temperature and fluid balance.

### Calculated variables

Calculated variables were obtained as previously described [11].

### Lung injury induction

After the baseline data collection, acute lung injury was induced with repeated whole lung lavage using one liter of isotonic saline (37°C) until the PaO_2_ was below 100 mmHg for at least 10 minutes. Lung injurious ventilation was then maintained for 30 minutes, the animal was ventilated in pressure controlled mode, with PEEP = 7 cmH_2_O, FiO_2_ = 1, inspiratory / expiratory time ratio (I:E) = 1:1, Ppeak = 42 cmH_2_O, and a RR of 20–30 breaths / minute. After 30 minutes, a second lavage with one liter of normal saline was performed, and the injurious ventilation was resumed. Arterial blood gases were obtained every 15 minutes at this step and the PEEP during the injurious ventilation was selected based on the PaO_2_ level. The injurious ventilation was interrupted if at least one of the following parameters was achieved:

- A period of 240 minutes after the second lung lavage;
- A mean pulmonary artery pressure > 50 mmHg;
- A static respiratory compliance < 10 mL/cmH_2_O (with a PEEP = 10 cmH_2_O and VT = 6 mL/kg);
- A PEEP persistently ≥ 15 cmH_2_O for at least two consecutive arterial blood sample analysis;
- A mean systemic blood pressure < 70 mmHg in spite of the use of norepinephrine in a dosage higher than 0.5 mcg/kg/min.

### PEEP adjustment

After the lung injury induction, a stepwise recruitment maneuver was performed with PEEP levels of 25, 35, and 40 cmH_2_O, a driving pressure (ΔP) of 20 cmH_2_O, RR = 10–20 breaths/min, and I:E ratio = 1:1. The first two PEEP levels were maintained for 40 seconds, and the PEEP = 40 cmH_2_O was sustained for two minutes. The recruitment maneuver was followed by a decremental PEEP titration while monitoring the amount of lung collapse with the EIT. The titration was done with steps of two minutes of PEEPs starting from 25 cmH_2_O and reducing 2 cmH_2_O in each step until an alveolar collapse >3% was reached. The ideal PEEP was considered to be 2 cmH_2_O higher than the value in which <3% of collapse occurred. This PEEP was considered to be related to an open lung approach (OLA), so it was called PEEP_OLA_.

**Fig 1:**
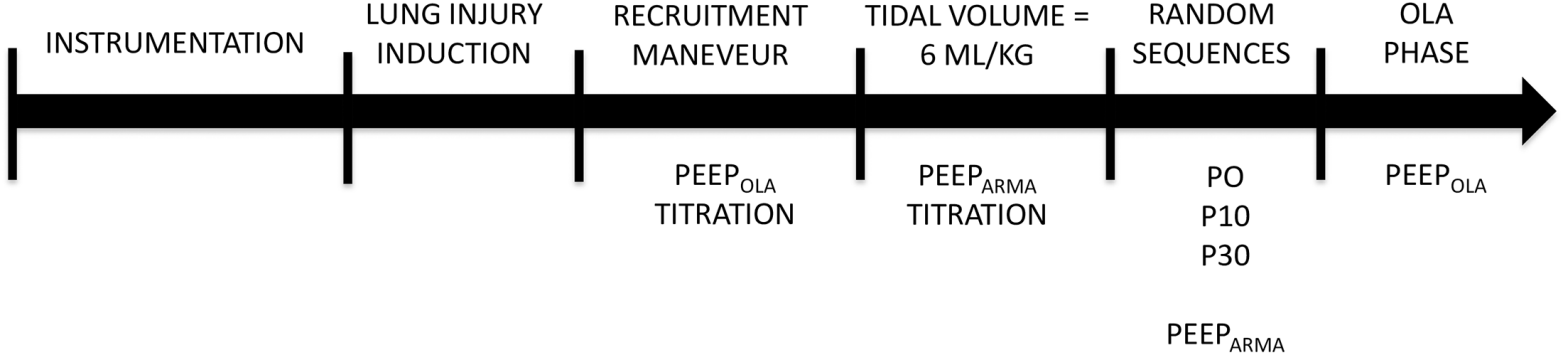
Timeline of the study. P0 denotes no inspiratory pause. P10 and P30 denote the static inspiratory pause of 10 and 30% of the inspiratory time respectively. OLA denotes open lung approach. PEEP ARMA denotes the positive end expiratory pressure titrated according to the PEEP table from the study [5].

Then, mechanical ventilation in volume-controlled mode was initiated, with VT = 6 mL/kg, RR = 35 breaths/min, PEEP = 10 cm H_2_O and FiO_2_ = 1. An arterial blood sample was obtained every ten minutes. In this stabilization step, PEEP and FiO_2_ were titrated according to the ARMA study PEEP table (aiming a PaO_2_ = 55–80 mmHg, and since it was titrated using a table, it was nominated PEEP_Table_ to differentiate from the PEEP_OLA_ (Fig 1) [5]. However, the tidal volume and RR were kept constant, in spite of PaCO_2_ and pH values, which one would expect to be shifted to an acidemia scenario.

### Experimental protocol

The PEEP_Table_ was used during P0, P10 and P30 to test the effect of different inspiratory pauses while breathing pure oxygen with a fixed RR of 60 breaths per minute and an inspiratory flow of 1 L/s or higher depending on whether inspiratory pauses were used. VT was adjusted to maintain PaCO2 levels within the target range of 57–63 mmHg. After every change in ventilatory parameters during the respective step of the study, we allowed CO_2_ to stabilize for at least 30 minutes drawing arterial blood gases every 10 minutes. We defined CO_2_ equilibrium as at least three consecutive PaCO_2_ measurements within the target range. In this initial random sequence, three different steps were tested in a randomized order using sealed envelopes: 1) pause = 0 (P0), 2) pause of 10% of the inspiratory time (P10), and 3) pause of 30% of the inspiratory time (P30).

After completion of the randomized sequence, the animals were submitted to an alveolar recruitment maneuver and ventilated with the same parameters as in the previous phase, except for PEEP (PEEP_OLA_) and VT (titrated to reach CO_2_ target), and with no inspiratory pause. This step (OLA) always occurred last, because of the higher PEEP as compared to the PEEP_table_ applied in the phase of random inspiratory pauses.

At each step, PEEPi was measured to adjust PEEPe aiming at PEEP_Total_ equal to PEEP_Table_ or PEEP_OLA_ according to the study phase. Between the different steps, a 40s disconnection from the ventilator was done to homogenize lung history. The timeline of the protocol is shown in Fig 1.

At the end of the experiments, the anesthesia was deepened with propofol overdose, and the animals were euthanized with a bolus of 10mL of potassium chloride 19.1%.

### Statistical analysis

The Shapiro-Wilk goodness-of-fit model showed a non-parametrical distribution in a large amount of variables; therefore data are reported as median (P25^th^, P75^th^). Wilcoxon`s Signed Rank Test was used to test variables before and after lung injury induction and to compare the upper and lower regional ventilation with the EIT. In order to avoid type I error, a modified Bonferroni’s correction was used to account for the multiple comparisons between upper and lower regions of ventilation. The analysis of variance for repeated measures on ranks (Friedman’s test) was used for analyses during the ventilatory modes tested. The *post-hoc* analyses were done using Student-Newman-Keuls’ test. A P < 0.05 was considered significant. The analyses and graphs were done with the Sigma plot 12.0 statistical package software (Systat Software, Inc. San Jose, California, USA).

## Results

In eight pigs weighting 34 [29,39] kg, lung injury was induced using 10 [7,16] liters of normal saline followed by injurious mechanical ventilation for 210 (19) minutes. As compared with baseline, lung injury led to a significant reduction in oxygenation (P/F ratio 92 [63,118] vs. 427 [368,473] mmHg, P < 0.01) and in the compliance of the respiratory system (11 [8,14] vs. 27 [15,30] mL/cmH_2_O, P < 0.01). The time to equilibrium of PaCO_2_ was similar in the different phases of the experiment and equal to 50 [40,65] minutes.

**Fig 2:**
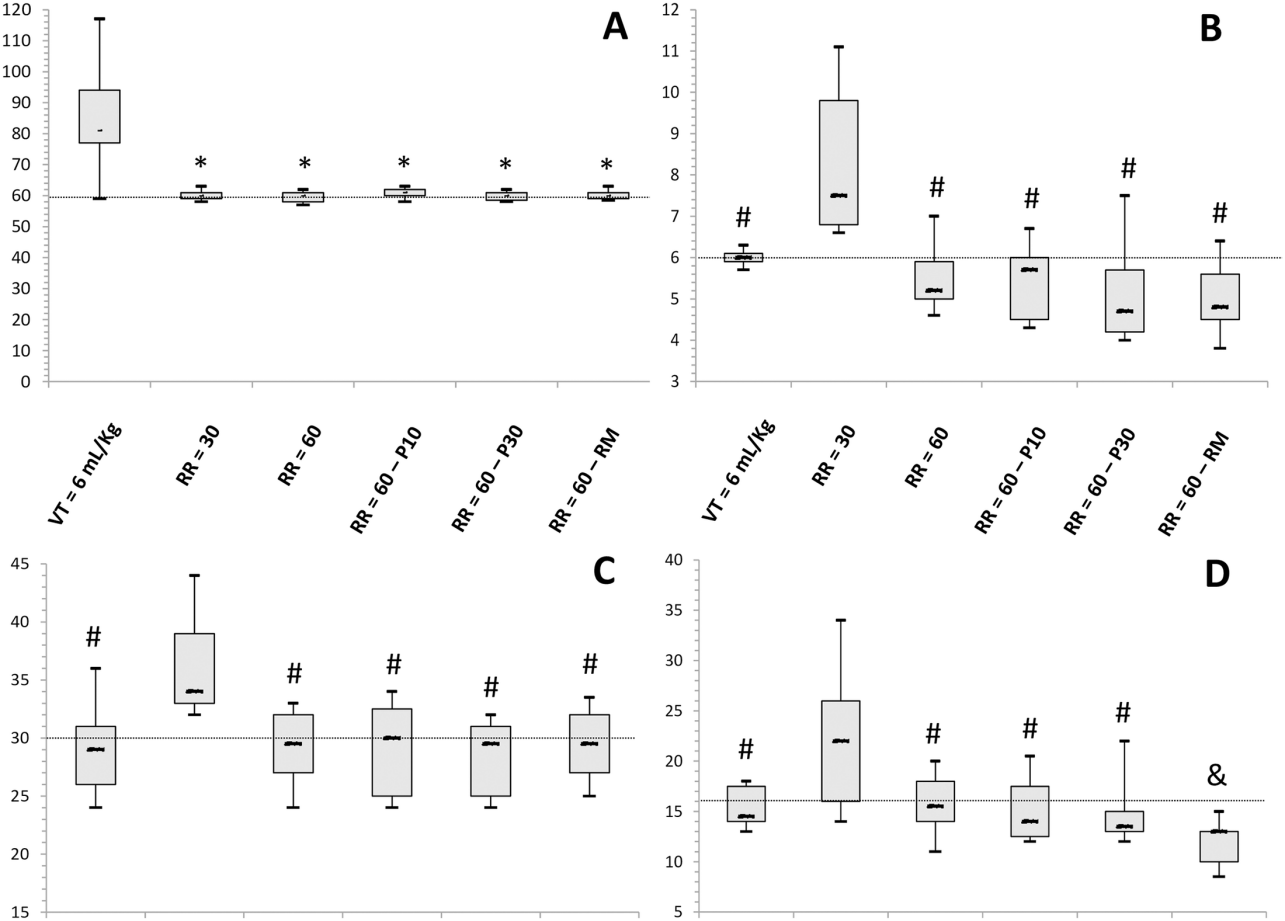
Respiratory variables during the ventilatory modes tested with eight animals. Panel A shows the PaCO2 variation during the experiments. Panel B shows the tidal volume per kg. Panel C shows the plateau pressure. Panel D shows the driving pressure. VT denotes tidal volume and RR respiratory rate. The whiskers denote the P10th and P90th. P0 denotes the phase without an inspiratory pause. P10 and P30 denote the static inspiratory pause of 10 and 30% of the inspiratory time respectively. RM denotes recruitment maneuver; * Student-Newman-Keuls’ post-hoc analysis, P < 0.05 vs VT = 6 mL/kg; & Student-Newman-Keuls’ post-hoc analysis, P < 0.05 vs all others.

PaCO_2_ was kept at the planned range of 57–63 mmHg (Fig 2A). After lung recruitment, both dead space and shunt decreased (Table 1) Tidal volume, however, was similar between study phases (Fig 2B).

**Table 1:** Respiratory variables of eight animals during the ventilatory modes studied.

Variable	RR = 60	RR = 60-P10	RR = 60-P30	RR = 60-OLA	P value^¶^
P/F ratio—mmHg	141 [105,184]	124 [90,179]	140 [110,162]	212 [191,243]*	P < 0.001
Gradient (A-a)O_2_	427 [384,466]	444 [389,479]	431 [409,458]	357 [326,378]*	P = 0.002
Minute ventilation—L/min	10.9 [10.6,12.0]	10.4 [9.0,11.4]	10.6 [9.0,12.2]	10.3 [9.7,10.7]	P = 0.091
Shunt—%	32 [27,49]	36 [28,52]	32 [28,43]	23 [20,23]*	P = 0.001
EtCO_2_—mmHg	41 [38,48]	46 (41, 51)	42 (36,48)	54 (52,60)*	P = 0.001
Vd/Vt—%	30 [20,37]	24 [14,33]	32 [10,46]	10 [0,15]*	P < 0.001
C_static_−mL/cmH_2_O	12 [10,15]	13 [10,16]	13 [11,14]	13 [13,16]*	P = 0.018
C_dyn_−mL/cmH_2_O	6 [5,7]	4 [3,4]*	4 [3,5]*	6 [6,8]	P = 0.001
Resistance—cmH_2_O/L/sec	14 [13,16]	18 [15,20]*	17 [13,18]	13 [12,14]	P = 0.014
PEEP total—cmH_2_O	14 [10,17]	15 [10,17]	14 [10,16]	17 [16,19]*	P = 0.005
PEEP intrinsic—cmH_2_O	0	0.5 [0,1]	0 [0,1]	0	P = 0.081
PEEP extrinsic—cmH_2_O	14 [10,17]	14 [10,17]	14 [10,16]	17 [16,19]*	P = 0.001
Peak pressure—cmH_2_O	39 [48,41]	53 [47,60]*	45 [41,55]*	39 [36,43]	P < 0.001
Mean airway pressure—cmH_2_O	18 [16,21]	19 [17,24]*	23 [21,26]*	22 [20,24]*	P = 0.002
Inspiratory flow—L/sec	1	1.69 [1.42,2.00]*	1.59 [1.34,1.76]*	1	P < 0.001
T_insp_/T_tot_—%	24 [20,30]	24 [19,56]	27 [19,40]	29 [20,54]	P = 0.969

RR denotes respiratory rate.

P10 and P30 denote the static inspiratory pause of 10 and 30% of the inspiratory time respectively.

OLA denotes open lung approach

Other abbreviations: C_stat_ and C_dyn_−static and dynamic compliance; EtCO_2_ –end tidal CO_2_; Gradient (A-a)O2 –Alveolar-arterial oxygen gradient; PEEP—positive end expiratory pressure and T_insp_/T_tot_−inspiratory time / total time of the respiratory cycle.

^¶^ The P value was obtained through the Friedman’s test

* Student-Newman-Keuls’ *post-hoc* analysis, P < 0.05 vs RR

Despite the higher PEEP_OLA_ as compared to that of other phases (Table 1), plateau pressures were similar in all four phases of the protocol (Fig 2C), which explains the lower driving pressures after lung recruitment (Fig 2D).

**Fig 3:**
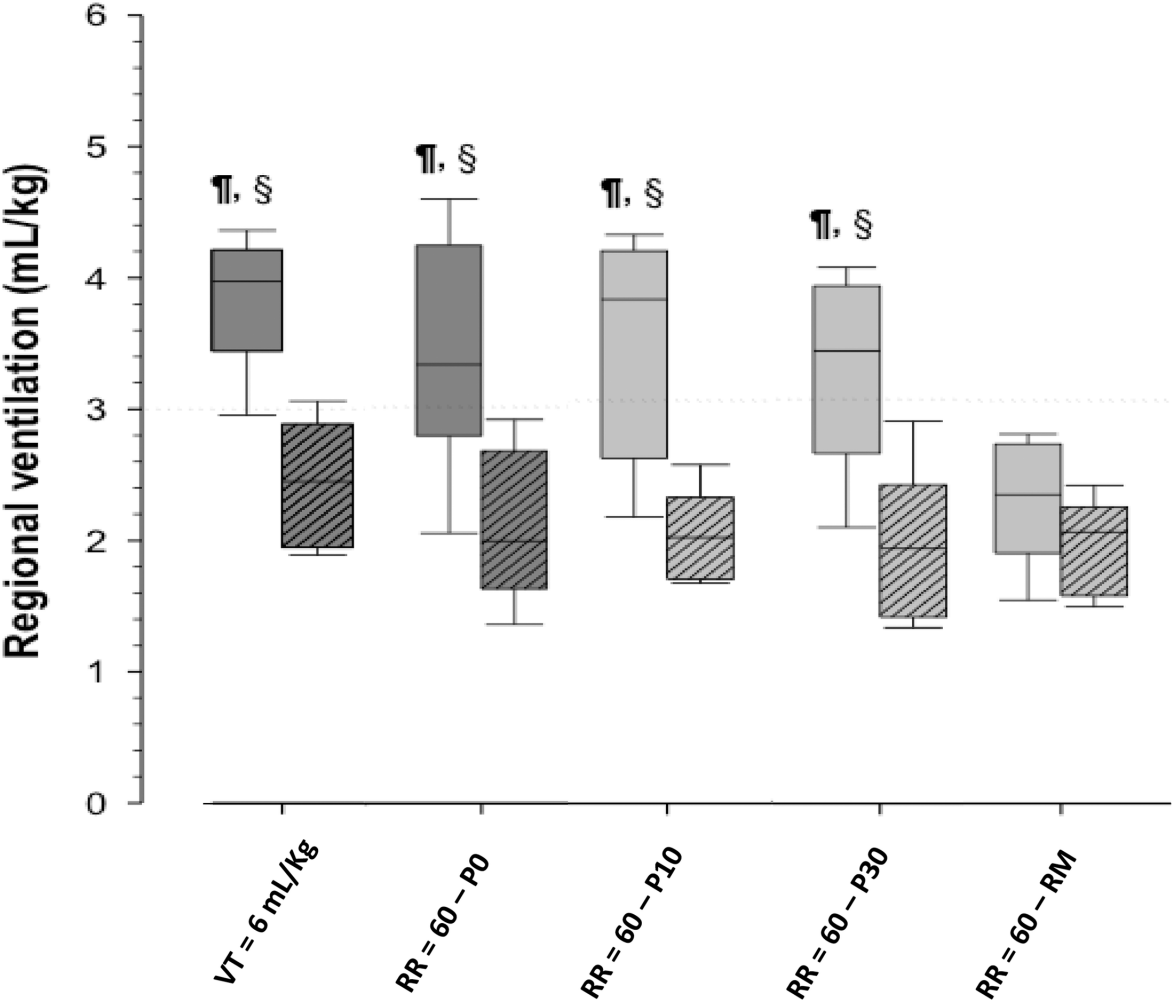
Regional ventilation (upper and lower distribution of air) of animals measured through the electrical impedance tomography during the ventilatory modes studied showing the tidal volume per kg of the upper and lower regions during the various frequencies studied. VT, RR, denote tidal volume, respiratory rate, respectively. P0 denotes the phase without an inspiratory pause. P10 and P30 denote the static inspiratory pause of 10 and 30% of the inspiratory time respectively. RM denotes recruitment maneuver. The whiskers denote the P10th and P90th. Hatched boxes are the gravitational dependent (lower) region. Non-hatched boxes are the non-gravitational dependent (upper) regions. § Wilcoxon`s test, P < 0.008 (Bonferroni’s correction for multiple comparisons) vs lower; ¶ Student-Newman-Keuls’ post-hoc analysis, P < 0.05 vs HFPPV = 60 w/ RM.

Ventilation distribution was asymmetric, with reduced ventilation to the dependent parts of the lungs, except during the phase after lung recruitment, in which the distribution was more homogeneous (Fig 3). This difference was statically significant.

The hemodynamic and metabolic data are shown in Table 2. Of note, pulmonary artery pressures and pulmonary vascular resistances were statically lower after lung recruitment.

**Table 2:** Hemodynamic and metabolic variables of eight animals during the ventilatory modes studied.

Variable	RR = 60	RR = 60-P10	RR = 60-P30	RR = 60-OLA	P value^¶^
**Hemodynamic**					
Heart rate—bpm	173 [153,181]	167 [146,188]	155 [128,182]	147 [138,166]	P = 0.030
Cardiac index—mL/kg/min	146 [120,169]	138 [112,163]	139 [122,150]	127 [116,155]	P = 0,829
SV—mL	27 [27,35]	26 [24,34]	30 [25,39]	29 [27,36]	P = 0.599
ABPm—mmHg	84 [74,94]	83 [73,107]	85 (20)	79 [68,110]	P = 0.927
PAPm—mmHg	34 [29,36]	35 [31,38]	35 [32,39]	28 [26,30]	P = 0.040
CVP—mmHg	8 [6,10]	9 [8,9]	9 [7,10]	10 [9,12]*	P = 0.006
PAOP—mmHg	12 [9,14]	12 [10,13]	12 [10,14]	12 [10,13]	P = 0.936
SVRI—dynes.s^-1^.(cm^5^)^-1^.kg^-1^	46 [36,50]	45 [35,59]	47 [39,61]	47 [31,60]	P = 0.930
PVRI—dynes.s^-1^.(cm^5^)^-1^.kg^-1^	13 [09,14]	12 [10,17]	13 [12,15]	09 [7,14]	P = 0.040
**Metabolic**					
Lactate—mEq/L	1.4 [0.7,1;4]	1.3 [1.0,1.6]	1.5 [1.0,1.7]	1.3 [0.8,2.1]	P = 0.942
pH	7.25 [7.23,7.34]	7.24 [7.22,7.28]	7.26 [7.23,7.35]	7.27 [7.22,7.31]	P = 0.801
Temperature–°C	39.4 [37.6,39.6]	39.2 [38.2,39.5]	39.1 [38.5,39.6]	39.7 [38.4,39.8]	P = 0.146
Fluid balance—mL	40 [-68,123]	180 [63,283]	75 [11,135]	33 [-13,81]	P = 0.412

RR denotes respiratory rate.

P10 and P30 denote the static inspiratory pause of 10 and 30% of the inspiratory time respectively.

OLA denotes open lung approach

Other abbreviations: ABPm—mean systemic arterial blood pressure; CVP—central venous pressure; PAOP—pulmonary artery occlusion pressure; PAPm—mean pulmonary arterial pressure; PVRI—pulmonary vascular resistance index; SV—strove volume; SvO2—mixed venous oxygen saturation and SVRI—systemic vascular resistance index.

^¶^ The P value was obtained through the Friedman’s test

* Student-Newman-Keuls’ *post-hoc* analysis, P < 0.05 vs RR = 6

## Discussion

Our findings showed that during protective mechanical ventilation of a severe ARDS swine model, MHFPPV using a conventional ventilator allows reaching tidal volume below 6 mL/Kg at clinically acceptable values of PaCO_2_. The recruitment maneuver associated with PEEP titration led to better oxygenation and homogenization of lung ventilation and allowed reduction in driving pressure. The addition of inspiratory pauses and/or recruitment maneuver with PEEP titration did not promote a tidal volume reduction significantly.

In bench models, a respiratory rate between 50–60 breaths/min was identified as the optimal frequency in ARDS regarding equilibrium between adequate alveolar ventilation using lower tidal volumes and peak inspiratory pressures [20]. This strategy, called mid-frequency ventilation, was tested using 6 pigs submitted to lung injury. With respiratory rates of 55.7±15.8 breaths/min the authors obtained the same minute ventilation as conventional ventilation but with lower VT (-1±0.7 mL/kg) with the same mean airway pressure but with higher auto-PEEP [21]. Experts in ARDS advocated that a strategy using higher respiratory rates than used conventionally can be physiologically appealing and should be more explored [22].

Our group has previously demonstrated that it is possible to apply MHFPPV using a conventional ventilator [11]. In that study, after testing different non-conventional respiratory rates (ranging from 60 to 150 breaths/min), we found that the physiologic benefits could be optimally achieved with a RR of 60 breaths/min. We therefore decided to focus on this respiratory rate and test other potential approaches that could be integrated to MHFPPV to promote additional physiological benefits, such as inspiratory pauses and recruitment maneuvers.

Astrom et al.[16] in a pig model of acute lung injury, showed that inspiratory pauses could enhance the CO_2_ elimination by prolonging the time available for gas distribution and diffusion within the respiratory tree, resulting in a reduction of the dead space [15], an effect even more important when associated with low tidal volumes [17]. The positive effects of this approach decreased when high initial inspiratory flows were present, which can explain our neutral results when MHFPPV was associated with an inspiratory pause [16]. This concept, however, had never been tested at very high RRs. It is possible that, under these circumstances, the prolongation of the mean distribution time of the inspired gas did not result in enough time for effective diffusion of CO_2_ towards the proximal airway, thus blunting the effect of the inspiratory pause on the CO_2_ elimination.

There are much uncertainties in the literature regarding the benefits and risks of recruitment maneuvers in ARDS patients [23]. Amato et al. demonstrated that a high PEEP titrated after an alveolar recruitment maneuver, associated with a low tidal volume ventilation was associated with decreased mortality in ARDS patients [4]. A meta-analysis showed that higher PEEP values were associated with a higher probability of survival when applied to more severe ARDS patients [24]. A mechanism by which higher PEEP values could reduce mortality is by promoting alveolar recruitment and increasing the functional size of the lung, resulting in lower driving pressures for a fixed tidal volume, as recently suggested [13].

Alternatively, PEEP could promote further tidal volume reductions by decreasing shunt and alveolar dead space [18]. In our study, we demonstrated that a strategy of a moderately-high respiratory rate (RR of 60 breaths/min) coupled with recruitment maneuver and PEEP titration was feasible and able to reduce both shunt and dead space ventilation thus improving gas exchange when compared to the other strategies testes using the same respiratory rate of 60 bpm. Additionally, it was associated with the lowest driving pressure values and a more homogeneous distribution of the ventilation.

Our study has several limitations. First, the arbitrary choice of the target CO2 level can be criticized. There are different options regarding the best CO2 target in ARDS patients, where some studies showing a protective [25] and others a potentially deleterious role [26]. However, the exact value chosen would hardly modify the main findings of the study. Second, with successive changes in the ventilator settings, a carryover phenomenon can potentially be expected. We tried to avoid that effect through the randomization of sequences, the disconnection from the ventilator between the steps, and through a prolonged wait to PaCO2 equilibrium. Third, we did not evaluate histological damage to the lungs that might have happened at higher RRs. Fourth, we might have been underpowered to detect small changes in tidal volumes. For example, direct comparison indicated lower tidal volumes in the OLA phase as compared to baseline, but the signal was too weak to affect the overall (Friedman) test of comparison between all phases. Finally, we cannot directly extrapolate these experimental findings to patients, whose time constants are different from those of pigs and might not tolerate very high RRs. Of note, a recent study in patients with ARDS showed that protective tidal-volumes around 4 ml/kg can be achieved with modest increments in RR, provided that care is taken to minimize the circuit dead-space [14].

## Conclusion

In an animal model of severe ARDS, MHFPPV set with a RR of 60 bpm delivered by a conventional ventilator in a severe ARDS swine model, inspiratory pauses did not allow reduction of tidal volumes. PEEP titration after a recruitment maneuver decreased driving pressures and improved both shunt and dead space ventilation, but not enough to lower tidal volumes significantly.
